# Investigating the Effect of Carbon Nanotube Diameter and Wall Number in Carbon Nanotube/Silicon Heterojunction Solar Cells

**DOI:** 10.3390/nano6030052

**Published:** 2016-03-22

**Authors:** Tom Grace, LePing Yu, Christopher Gibson, Daniel Tune, Huda Alturaif, Zeid Al Othman, Joseph Shapter

**Affiliations:** 1 Centre for Nanoscale Science & Technology (CNST), Flinders University of South Australia, South Australia 5042, Australia; tom.grace@flinders.edu.au (T.G.); leping.yu@flinders.edu.au (L.Y.); christopher.gibson@flinders.edu.au (C.G.); daniel.tune@flinders.edu.au (D.T.); 2 Institute of Nanotechnology, Karlsruhe Institute of Technology, 76021 Karlsruhe, Germany; 3 Advanced Material Research Chair, Chemistry Department, College of Science, King Saud University, Riyadh 11451, Saudi Arabia; h.a.alturaif@hotmail.com (H.A.); zaothman@ksu.edu.sa (Z.A.O.)

**Keywords:** carbon nanotubes, solar cells, carbon nanotube (CNT)/Si heterojunction solar cells, double-walled carbon nanotube (DWCNT), multi-walled carbon nanotubes (MWCNT)

## Abstract

Suspensions of single-walled, double-walled and multi-walled carbon nanotubes (CNTs) were generated in the same solvent at similar concentrations. Films were fabricated from these suspensions and used in carbon nanotube/silicon heterojunction solar cells and their properties were compared with reference to the number of walls in the nanotube samples. It was found that single-walled nanotubes generally produced more favorable results; however, the double and multi-walled nanotube films used in this study yielded cells with higher open circuit voltages. It was also determined that post fabrication treatments applied to the nanotube films have a lesser effect on multi-walled nanotubes than on the other two types.

## 1. Introduction

The search for efficient, low-cost renewable energy sources is one of great importance in the modern world. As the conventional fossil fuel sources of electricity become scarcer, and are discovered to cause severe problems for our planet’s climate, it becomes all the more imperative to seek out new and innovative ways of exploiting sustainable resources.

Solar energy is the quintessential sustainable resource, however there are some significant disadvantages of current commercial solar cells. Firstly, silicon solar panels have a high manufacturing cost due to the need for high purity, processed silicon to produce high efficiency solar panels. While other semiconducting materials may be used in photovoltaic (PV) devices, they will generally consist of alloys of rare and/or toxic elements such as arsenic, cadmium, indium, gallium, germanium and ruthenium [1]. Innovations in PV technologies are making solar cells more and more competitive. Some methods of improving the economic viability of solar capture technology involve investigating solar cells containing organic molecules, quantum dots, or dye-sensitized solar cells [2]. However, this paper investigates a cell design that combines the already established good solar properties of silicon with an inexpensive material, namely carbon nanotubes (CNTs). Carbon nanotubes are considered to be an exciting prospect for solar cell integration due to a wide range of unique and interesting properties, such as; high charge carrier mobilities, ballistic transport properties, high optical transmittance, low light reflectance [3,4,5], and photoelectrochemical effects under light irradiation [3,5,6,7,8,9]. In addition, there are many different types of nanotubes to choose from with a wide array of possible bandgaps, thus the bandgap can be “tuned” for the situation. CNTs are also highly resistant to damage, whether it be mechanical, chemical or radiation induced [7]. Coupled with their potential low cost due to the abundance of carbon [4] and the miniscule quantities of material required in applications, it is clear why they are an exciting material for research. Solar cells consisting of a heterogeneous junction (heterojunction) between a (generally n-doped) silicon substrate and a CNT film have been shown to be an appealing prospect for the future of solar energy capture technologies.

In 2007, Wei *et al.* designed a new kind of cell in which CNTs function not only as transport charge carriers, but also assist in exciton separation [10,11,12,13], using double-walled CNTs (DWCNTs) deposited via water expansion and aqueous film transfer of an as grown chemical vapor deposition (CVD) film [11,12]. While these cells had a power conversion efficiency of only 1.3% (compared to commercial silicon cells at generally 13%–25%) [14,15] many improvements have since been made to the cell design and doping methods, with 15% efficiencies reported in 2012 [2] and 17% efficiencies reported in 2015 [6,16]. Although, for the most part, these efficiencies could only be achieved via the use of an anti-reflection coating on the surface of the solar cell, with the 15% value achieved by depositing a TiO_2_ layer over a solar cell which previously achieve efficiencies of around 8% and the 17% efficiency was achieved through the use of a molybdenum oxide layer on a solar cell design with an intrinsic efficiency of 11.1%. In terms of solar cell intrinsic efficiencies, without an anti-reflection layer, the highest in 2013 was 11.5% [17], which was improved to a record high intrinsic efficiency of 13.85% in 2015 [18]. Thus, in less than a decade the cell efficiency has been improved by a factor of 10. This rapid improvement is one of the reasons for much excitement around this solar cell design. In addition, these cells are interesting for future research as their manufacturing process is both simple and scalable [10]. The typical architecture for these solar cells is much like that of a single junction crystalline silicon solar cell with the emitter layer replaced by a film of p-doped CNTs [11]. While Wei’s initial design used DWCNTs, most studies have since used single-walled carbon nanotubes (SWCNTs), and multi-walled carbon nanotubes (MWCNTs) may also be used [10,19]. In all of these cases, the CNT film acts as a component of the heterojunction to enable charge separation, as a highly conductive network for charge transport and as a transparent electrode to allow good light illumination and charge collection [10].

Li *et al.* [20] found that post-fabrication treatment of a SWCNT layer with the *p*-type dopant thionyl chloride (SOCl_2_) increased the power conversion efficiency of the cells by over 45% by lowering the sheet resistance, and increasing the short circuit current density (*J*_SC_) and open circuit voltage. Hall Effect measurements showed that the SOCl_2_ treatment led to an increase of carrier density from 3.1 × 10^15^ to 4.6 × 10^17^ cm^−2^ and an improvement from 0.23 to 1.02 cm^2^ V^−1^ s^−1^ of the effective mobilities [11,20]. In addition, it was found that SOCl_2_ treatment adjusted the Fermi level and shifted the major conduction mechanism in the SWCNT layer from hopping towards tunneling [21,22].

Jia *et al.* [23] performed the first comparison between SWCNTs, DWCNTs and MWCNTs for use in CNT/Si cells in terms of area density of the films. It was found that SWCNTs are superior to MWCNTs at low densities and that the density (and thus optical transmittance) is vitally important in the performance of these cells. Increasing film transparency (lowering CNT density) increases the efficiency of the cells by allowing more light to reach the silicon, while decreasing the transparency (increasing the CNT density) increases the efficiency by lowering the sheet resistance across the film [11,23]. Thus, some optimal thickness must exist to achieve maximum efficiency. This research team also found their DWCNT cells to be significantly superior to both the SWCNT and MWCNT cells. However, their DWCNT films were produced using a different method to the SWCNT and MWCNT films [23] and were significantly longer and more pristine. This makes it difficult to draw a good comparison, as the nanotube film properties are highly dependent on film morphology [11] and fabrication route.

The aim of the research reported in this paper was to provide a more reliable comparison by creating suspensions of single, double and multi-walled carbon nanotubes under the same solvent conditions and examining the differences in solar cell properties between the different types. This study further improves on previous nanotube comparisons by using nanotubes that were specifically chosen to be of similar length and the films were all produced using the exact same procedure.

## 2. Results

**Figure 1:**
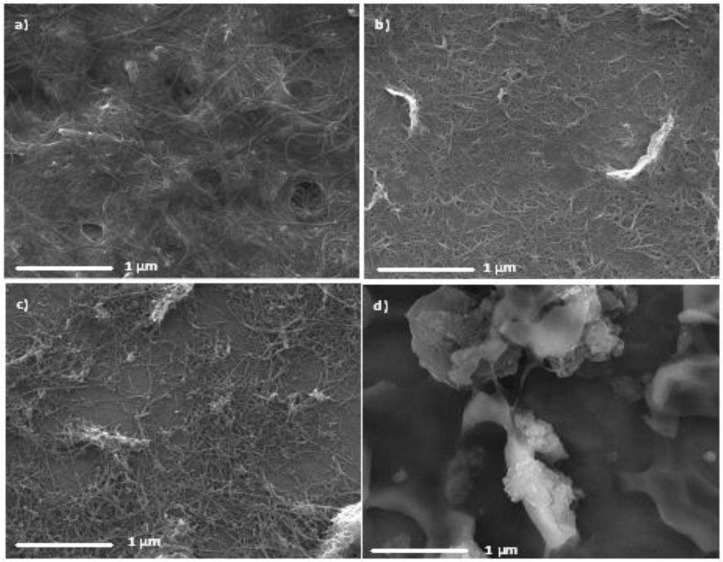

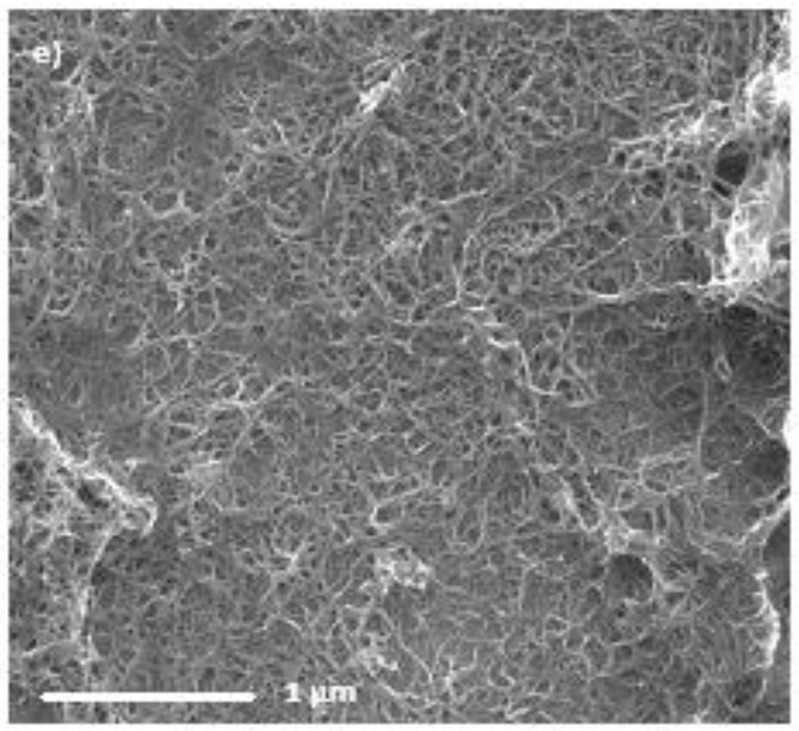
Scanning electron microscopy (SEM) images of various types of carbon nanotube (CNT) samples on Si: (**a**) single-walled carbon nanotube sample 1 (SWCNT-1); (**b**) single-walled carbon nanotube sample 2 (SWCNT-2); (**c**) double-walled carbon nanotube sample 1 (DWCNT-1); (**d**) DWCNT-2; and (**e**) multi-walled carbon nanotube (MWCNT).

Films were produced for each nanotube sample and were deposited on silicon substrates and imaged with scanning electron microscopy (SEM) to determine the film morphology. The images are shown in Figure 1. It is immediately noticeable that the DWCNT-2 suspension did not form a homogeneous film as the SEM image shows that the nanotubes clump together rather than spread across the membrane during film formation. The poor film morphology is due to issues dispersing DWCNTs in suspension. There are several possible reasons for this poor dispersion including tube length, contaminants and the surface properties of the nanotubes in the sample. Due to the poor film morphologies and coverage obtained for the DWCNT-2 sample, further work with this sample proved unfruitful and thus it will not be discussed in the rest of this study. The DWCNT-1 sample also did not form a fully homogenous surface covering film, however the film coverage was much closer to the SWCNT and MWCNT samples than DWCNT-2. Due to difficulties faced in suspending the DWCNTs, those suspensions were more dilute than the SWCNT or MWCNT suspensions, despite the addition of the same volume of dry nanotubes. It may be that the DWCNTs tend to remain bundled together more in suspension than the other nanotube types and thus form less homogeneous, more clustered films. There does not appear to be a large difference in the film morphology between the SWCNTs and MWCNTs: all three of these samples appear to form good, homogeneous coverings. There are noticible holes in the SWCNT-1 films, which were formed during the vaccuum filtration process when the vacuum was allowed to run for a time after all liquid has passed through. These are likely more prominent in this example as the film formed was thicker than the others. Overall, the SEM images show that all the types of nanotubes form suspensions well enough to produce homogenous films on a substrate and are thus usable for PV solar cell projects.

Optical absorption spectroscopy was performed on the nanotube films to determine the thickness and was also performed on the nanotube solutions to help confirm the nanotube types. It is expected that the ultra-violet/visible (UV/Vis) spectrum for MWCNT and DWCNT nanotubes will be featureless with an increase in absorption as the light wavelength decreases. A different spectrum is expected for SWCNT nanotubes, for which the van Hove singularities present in the SWCNT density of states lead to characteristic peaks in the optical spectra.

**Figure 2:**
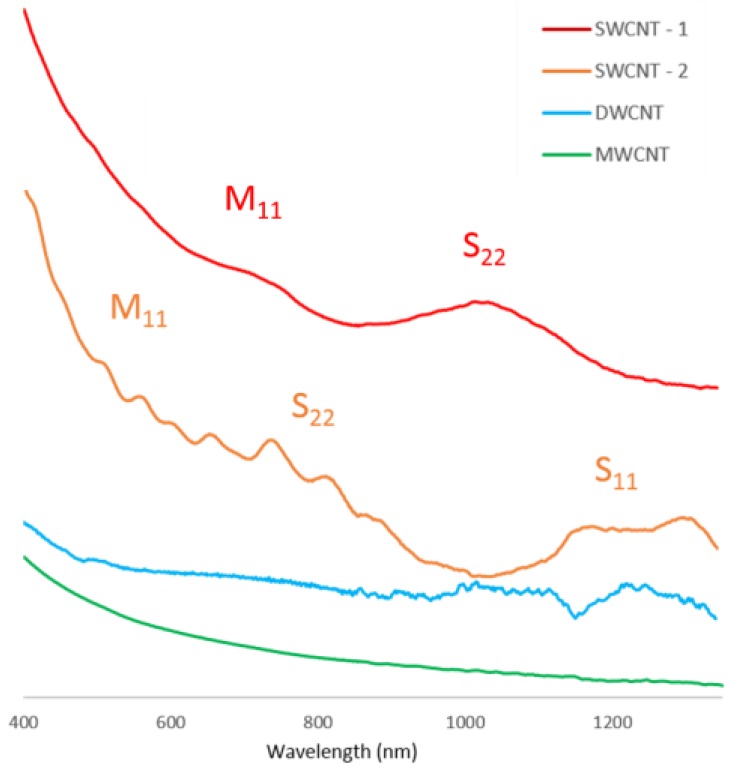
Ultra-violet/visible (UV/Vis) absorption spectra for all as prepared CNT suspensions. The absorption values have been offset to allow easier viewing. Included in the figure are reference labels for semiconducting tube Van Hove singularity transitions S_11_ and S_22_ and metallic tube Van Hove singularity transition M_11_.

It can be observed from Figure 2 that the SWCNT spectra display peaks in the regions of 600–800 nm due to the S_11_ transition and 350–500 nm regions due to the M_11_ transition, and thus show the presence of SWCNTs in these solutions. This is the expected shape for UV/Vis spectra of single-walled nanotubes, due to their unique density of states allowing electronic transitions in the UV/Vis range. The differences in peak position in the two SWCNT samples are likely caused by species of different chirality being present in each sample. It is also noticeable that the SWCNT-1 sample had much less distinct peaks than the SWCNT-2 sample. This is likely due to the presence of a range of closely related nanotube chiralities in sample SWCNT-1. The spectra of the DWCNT and MWCNT samples are relatively featureless with an increase in absorbance at the lower wavelength end of the spectra as expected.

**Figure 3:**
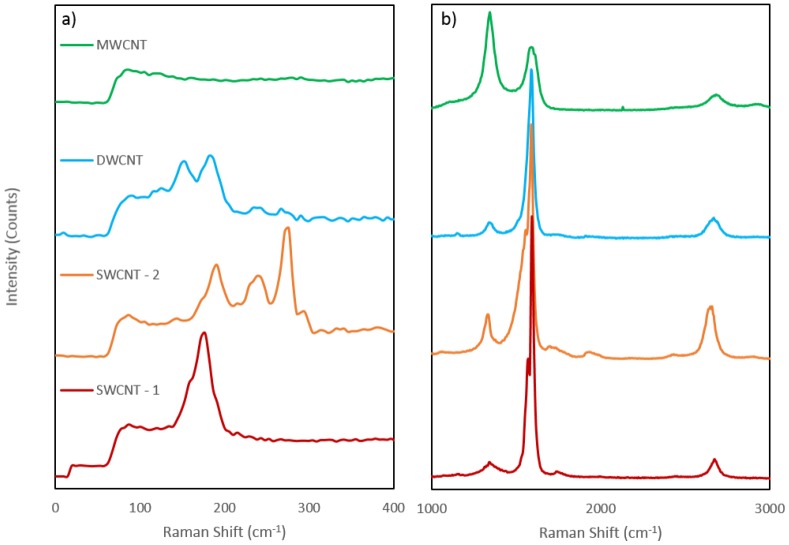
(**a**) Low wavenumber region of the Raman spectra for all CNT samples showing the D, G, G′ and Breit-Wigner-Fano (BWF) bands, the intensity values have been offset to allow for easier viewing. (**b**) High wavenumber region of the Raman spectra for all CNT samples, showing the radial breathing mode (RBM) region. The intensity values have been offset to allow for easier viewing.

In Raman spectroscopy, carbon nanotubes display characteristic peaks at around 1580 cm^−1^ and at around 1350 cm^−1^ known as the G and D bands, respectively. The G band is characteristic of highly ordered carbon species such as graphite or CNTs while the D band is characteristic of disordered carbon species. Thus the intensity of the G band relative to that of the D band is a strong indicator of crystallinity [24]. A radial breathing mode (RBM) between 100 and 500 cm^−1^ can also be uniquely observed in Raman spectra of carbon nanotubes and is seen as direct evidence for the presence of SWCNTs [25] or DWCNTs [24] in the sample. An overtone of the D mode, known as the G′ band (an overtone of the D band) is from a two-phonon, second-order Raman scattering process and expected to be seen at around 2700 cm^−1^ while a signal at 1550 cm^−1^ is known as a Breit-Wigner-Fano (BWF) band and is attributed to metallic carbons (metallic nanotubes in this case) [25]. Figure 3 displays the spectra obtained for each nanotube sample. As well as the characteristic D and G bands for CNTs the spectra also display the G′ band at 2700 cm^−1^ and while no RBM can be observed in the MWCNT sample. Peaks between 100 and 500 cm^−1^ can be observed in all other samples in Figure 3a. The ratio of the intensities of the D and G bands (D/G ratio) is often used to measure the disorder in carbon nanotube samples. The D/G ratio in the MWCNT sample is significantly different than that of the other four samples, as expected for MWCNTs [24]. The D/G peak height ratios for all nanotube types are shown in Table 1. It can be seen from the values in Table 1 that the SWCNT-1 and DWCNT-1 nanotubes exhibit the lowest ratios, and are thus the most highly ordered nanotube or purest species in this study. The SWCNT-2 sample exhibited a higher D/G ratio, indicating a higher level of disorder or impurity, where as the MWCNT sample gave a ratio above 1, due to the D peak being higher than the G peak, which is expected for MWCNTs [24,25]. The amount of CNT disorder is important to investigate as chemical reactions with nanotubes are more likely to occur at disordered regions than ordered regions.

The Raman shift at which the RBM occurs can be used to calculate the nanotube diameter for SWCNTs as the RBM frequency is inversly proportional to the diameter of the tubes [26]. The equation relating the RBM shift to the diameter is: $\text{RBM shift}\;\left(cm^{-1}\right)=\frac{A}{d_{t}\left(nm\right)}+B$ where A has an approximate value of 234 nm·cm^−1^ as determined from first principles (*ab initio*) calculations and B is approximately 10 cm^−1^, which corrects the intertube interaction frequencies in SWCNT bundles [26,27]. This equation is applied to the RBM data shown in Figure 3a to give a calculated diameter for the SWCNT-1, SWCNT-2 and DWCNT nantube samples and is compared with the manufacturer supplied diameters in Table 2 (note that the value of B was 0 in the DWCNT case as B is a correction for SWCNT bundles). Firsty, the calculation shows a very good agreement with the manufacturer supplied diameters for both of the SWCNT samples, with the calculated value(s) falling within the range given by the manufacturers. The DWCNT calculation does not agree, with the large peaks giving diameters around 0.75 nm smaller than the lowest diameter given by the manufacturer. This discrepancy can be explained by assigning the visible RBM peaks to the inner tubes of the DWCNTs since the interwall difference is known to be around 0.33–0.41 nm [28,29]. To achieve a diameter of >2 nm the RBM peak for the outer tube would have to be at or below 117 cm^−1^. The two small peaks to the left of the larger peaks on the DWCNT spectrum are closer to this range, with the left most peak occuring at around the expected 117 cm^−1^ and thus these smaller peaks could be due to the outer tubes.

A series of films of different transparencies were prepared to determine suspension volume required to give similar transmittances. The data from these experiments are provided in Table S1. Films of each nanotube sample were produced at approximately the same transmittance for use in the solar cells. All samples displayed good transmittance values between 58% and 65%.

The sheet resistances of the nanotube films at each stage of treatment are shown in Table 3. The data show that the SWCNT films had a lower sheet resistance than the DWCNT and MWCNT films. Since the amount of material in the films was the same, as indicated by the optical measurements, this suggests improved charge carrier transport in the SWCNT films. The changes in sheet resistance with treatment are expected and are due to carrier doping and reductions in tube-tube contact resistance.

### 2.1. Solar Cell Performance

Films of all nanotube samples were deposited on the standard solar cell substrates described in the experimental details and their solar cell performance was measured four times: once directly after fabrication and then once after each treatment (HF etch, SOCl_2_ treatment, and 2nd HF etch).

**Figure 4:**
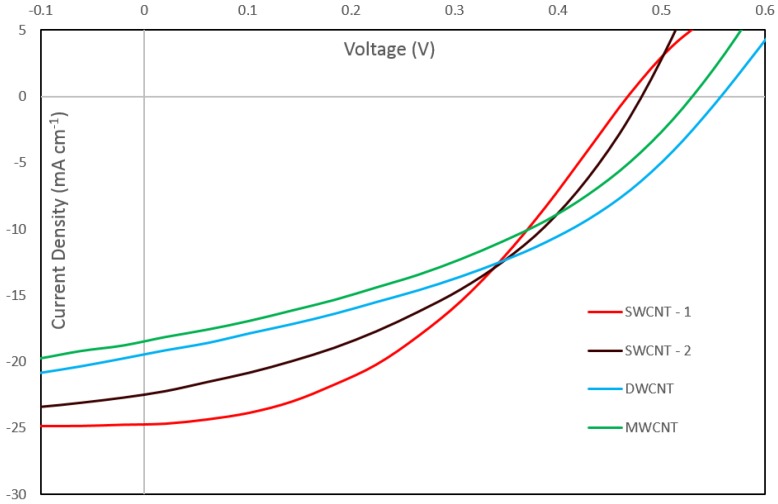
Current density *vs* voltage (*J*/*V*) curves for best performing cells for each CNT sample after the second HF etch. Curves for two single-walled carbon nanotube samples (SWCNT-1 and SWCNT-2), one double-walled carbon nanotube sample (DWCNT) and one multi-walled carbon nanotube sample (MWCNT) are shown.

Figure 4 shows the current-voltage characteristics of the best performing cells for each nanotube type after the complete treatment sequence, and the corresponding dark curves are shown in Figure S1. Both SWCNT samples show similar behavior under illumination, the only difference being a higher short-circuit current density (*J*_SC_) for SWCNT-1 and a slightly higher open circuit voltage (*V*_OC_) for SWCNT-2. Both the DWCNT and MWCNT samples display lower *J*_SC_ values. However, it can be seen that the DWCNT and the MWCNT cells produced a significantly higher *V*_OC_ than all the other cells. This is unexpected as the *V*_OC_ is determined by the energy levels of the system and should not be greatly affected by a change in nanotube type in this regard. It is likely, however, that this higher *V*_OC_ is caused by a lower rate of recombination in these nanotube types.

**Figure 5:**
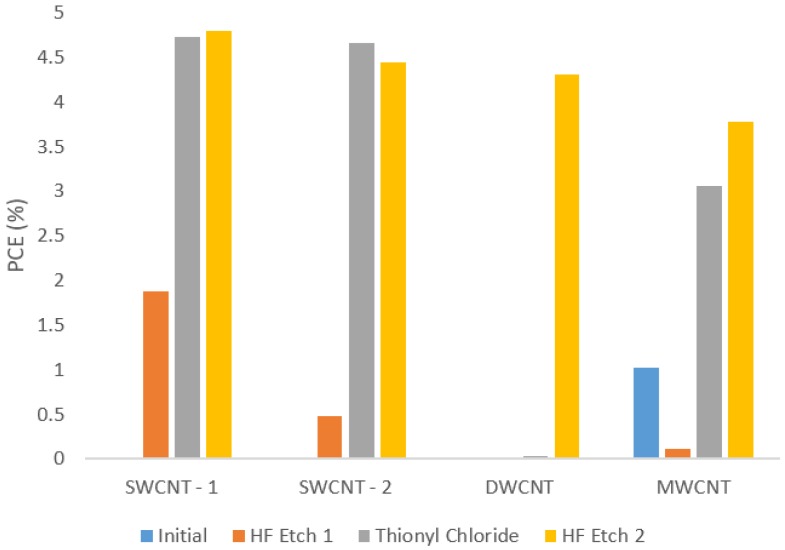
Solar cell efficiencies (%) for all nanotube types with treatment. PCE: power conversion efficiency.

Figure 5 shows how the power conversion efficiency (PCE) of the best performing cell of each nanotube type varies with treatment. By the second HF etch the best cell PCEs ranged from 3.78% for the MWCNT sample to 4.79% for the SWCNT-1 sample. This difference of 1% absolute, or over 25% relative (based on the MWCNT device), between the two nanotube types is quite significant considering the care taken to control other differences between devices and the fact that the average relative error was around 15%. The ratio of the direct current (DC) electrical to optical conductivity, σ_DC_/σ_OP_ for all films is very similar (see Table S1). The value is slightly higher for the SWCNT-1 film, which likely explains its higher PCE value. The origin of the large increase in performance of the DWCNT devices after the 2nd HF treatment is unknown. It is not consistent with a model in which the SOCl_2_ is the *p*-type dopant increasing conductivity and lowering Fermi energy relative to the silicon, and thus improving device performance, as this effect should be observed after the SOCl_2_ treatment. It could possibly be due to acid digestion of the non-nanotube carbonaceous impurities (up to 40% by weight in the starting material) by the sequence of aggressive chemical treatments, resulting in better contact between the (purer) nanotube film and the underlying silicon, but further investigations are required to shed light on this.

Cells containing MWCNTs generally performed better at the initial stage of testing, but did not improve to the same extent as the SWCNT and DWCNT cells with doping. This can be explained by considering the surface area to volume ratio of MWCNTs compared to SWCNTs and DWCNT. Multi-walled nanotubes have a larger bulk than the other types for the same surface area, and thus surface acting treatments such as HF and SOCl_2_ will not have the same effect on bulk MWCNT films. Additionally, unlike SWCNTs, the large diameter and complex mixing of wall types and energy states in MWCNTs means they only have metallic character and therefore there are no tube-tube energy barriers to be lowered by doping. Thus, the MWCNTs are less affected by the acid and the thionyl chloride treatments used in this study.

## 3. Discussion

Table 4 shows a breakdown of the solar cell data. It can be seen that the highest *J*_SC_ was achieved by the cell that gives the highest efficiency. This is expected, as the efficiency is proportional to the current density. The efficiency is also proportional to the open circuit voltage (*V*_OC_) and the fill factor (FF). Thus the fact that the SWCNT-1 sample also achieved the equal highest fill factor is expected. It is surprising that the open circuit voltage is lower for both SWCNT samples than for the DWCNT and MWCNT samples. The higher voltages for these samples can be attributed to the smaller saturation current (*J*_SAT_) values for these cells. A smaller *J*_SAT_ indicates less current flowing in the undesired direction in the cell. The relation between *J*_SAT_ and *V*_OC_ for the best performing cells for each sample can be seen in Figure S2. The huge error values attached to the *J*_SAT_ measurements are due to the order of magnitude differences between the *J*_SAT_ values in each cell for each sample. The improved efficiencies seen in the SWCNT samples over the DWCNT and MWCNT samples can be partially understood by observing the difference between the estimated shunt resistance (*R*_shunt_) and series resistance (*R*_series_). The series resistance value represents the amount of resistance opposing current flow in the desired direction and thus the ideal situation for a solar cell is to have a low *R*_series_. Conversely, the shunt resistance value represents the amount of resistance opposing current flow over “short-cuts” in the cell circuit, thus the ideal value for *R*_shunt_ is high. Table 4 shows that the difference between *R*_shunt_ and *R*_series_ is an order of magnitude in the SWCNT cases, but is less than this in the DWCNT and MWCNT cases. This is a likely explanation for the slightly improved PCE for the SWCNT samples. The SWCNT-1 sample produced a higher diode ideality than the other three samples, given that the ideal value is 1 it is apparent that this cell was not as ideal as the other samples. This is likely indicative of a poorer contact with the silicon substrate in the solar cell, when compared with the other samples.

Overall the SWCNT samples performed better than the other nanotube types. This is expected from the sheet resistance measurements, as the SWCNT samples exhibited lower resistance after the final treatment. This improved resistance is likely due to the better morphology of the SWCNT films compared to the DWCNT films. There has been some work to suggest that DWCNTs should conduct charge better [30]. The fact that the DWCNT sample produces a poorer film is likely traceable to the fact that the suspension is more difficult to make and the lower purity of most DWCNT samples. The MWCNT sample showed a good film morphology and overall good sheet resistance. However the resistance of these films did not decrease with treatment at the same rate as for the SWCNT films.

## 4. Materials and Methods

Five nanotube samples were used in this study, as shown in Table 5.

Each nanotube suspension was produced in the same manner. The nanotubes (95 mg) were added to an aqueous TritonX-100 solution (1% *v*/*v*, 50 mL) (Sigma-Aldrich, St. Louis, MI, USA) and bath sonicated (≈50 W_RMS_ (root mean squared Watts), 3 × 20 min intervals, Elmasonic S 30H (Singen, Germany). In between each sonication the sonicating bath water was changed to prevent the suspension heating too much during sonication. The resulting sonicated suspension was centrifuged (1 h, 17,500 g, Beckmann-Coulter Allegra X-22 (Brea, CA, USA) then the upper two thirds of the liquid in each tube was carefully extracted, combined, and centrifuged again with the same parameters. The upper two thirds of the liquid in each tube was carefully extracted and combined. The remaining third contained black lumps of unsuspended carbon and was discarded.

**Figure 6:**
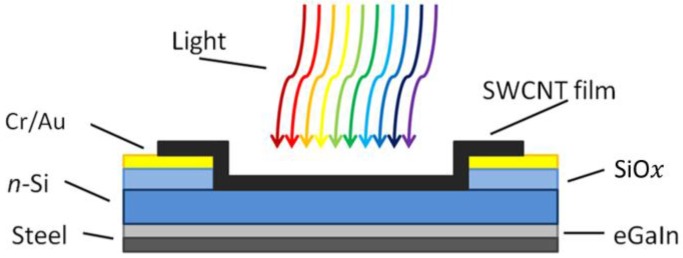
Simple schematic of the cells used in this experiment (Not to scale). Gallium indium eutectic (eGaIn).

The photovoltaic cell substrates were produced by cutting silicon pieces of approximately 1.5 cm^2^ from a wafer of n-doped (phosphorous) silicon with a 100 nm oxide layer on one side. Each piece was patterned using a positive photoresist (Methoxypropyl acetate) applied via spin coating (30 s, 3000 rpm) and soft baked (100 °C, 1 min). The resist was exposed to UV light through a mask and the exposed resist was dissolved in a developer solution (trimethyl ammonium hydroxide) to leave an active area of 0.079 cm^2^ still covered. The substrate was sputter coated with a 5 nm layer of chromium and a 145 nm layer of gold. The thickness is controlled by a quartz crystal microbalance. The gold coated substrates were submerged in acetone (30 min) before sonicating briefly to dissolve the unexposed resist. One drop of buffered oxide etch (BOE, 6:1 ratio of 40% NH_4_F and 49% hydrofluoric acid (HF)) was placed on the active area for 90 s in order to remove the 100 nm thermal oxide and allow the nanotubes to contact the silicon [31]. A schematic of the photovoltaic cell substrate is shown in Figure 6.

The nanotube films were produced via vacuum filtration. This was accomplished by first mixing the required amount of nanotube suspension (dependent on the concentration of the suspension in question, can vary from tens of microliters to up to 15 mL) with MilliQ water (Kansas City, MO, USA) to produce a solution of 250 mL. This solution was filtered using a vacuum produced with a water aspirator through a series of two microporous filter papers. The bottom filter paper (VSWP, Millipore (Billerica, MA, USA), 0.025 μm pore size) was patterned with holes the size of the desired nanotube films (0.5 cm^2^ in this case), while the top filter paper (HAWP, Millipore (Billerica, MA, USA), 0.45 μm pore size) was unpatterned. The difference in flow rate through the filter papers causes the solution to pass preferentially through the top film where the bottom film is patterned. The nanotubes are thus caught by the top film in the same shape as the template film. The solution that had passed through both films was passed through the filtration apparatus two more times, to ensure enough nanotubes were retained on the top film. Pure MilliQ water was then passed through to wash out Triton X-100 surfactant remaining in the nanotube film. In most cases in these experiments the template used in each filtration produced four 0.5 cm^2^ films, one for attachment to glass to measure the optical transmittance, sheet resistance and Raman spectra and the other three for attachment to solar cells.

Nanotube films were attached to either glass (prewashed in ethanol) or the silicon substrate in the same manner. The films were cut from the filter paper and placed nanotube side down on the substrate. They were wetted with a small drop of water and sandwiched with a piece of Teflon and a glass piece and clamped together. The clamped substrate was heated at around 80 °C for 15 min then left to cool in the dark for 1 h. The substrates were washed in acetone (first wash 30 min, second and third wash 30 min with stirring) to remove the attached filter paper. To complete cell preparation, the reverse side of each piece of silicon was manually scratched to remove the oxide layer. A gallium indium eutectic (eGaIn) was applied to the scratched surface and then a piece of stainless steel was attached as the back contact.

The cells were tested after the steel attachment and then a further 3 times after different post fabrication treatment steps. Firstly a drop of 2% HF was applied to the active area to etch silicon oxide formed between the silicon and the nanotube film produced during the film attachment step. Secondly, the nanotube film was treated with a few drops of thionyl chloride (SOCl_2_) and left until it evaporated, the residue was washed with ethanol prior to testing. The last step was another 2% HF treatment, which is observed to significantly improve performance, though the mechanism for this is still unclear [32].

At each stage of treatment the solar cells were tested by applying contacts to the back and front electrodes and reading the current density as the voltage is ramped from 1 V to −1 V. This is performed both in the absence of light and under illumination provided by a solar spectrum simulator at 100 mW·cm^−1^ to measure both a “dark” and “light” curve the light is filtered through an AM1.5G filter (Irvine, CA, USA). The irradiance at the sample was kept constant by measuring with a silicon reference cell (PV Measurements, National Institute of Standards and Technology (NIST)-traceable calibration). The information was collected using a Keithley 2400 SourceMeter (Solon, OH, USA) attached to a PC running a program written in LabView (Austin, TX, USA).

The amount of light that passes through the CNT film to reach the CNT/Si junction directly affects the amount of energy that the cell can produce. Thus, it was important to perform nanotube type comparisons between films that allowed a similar amount of light to pass through. A series of films were produced from each sample using different amounts of nanotube suspension. The average light transmittance over a wavelength range of 300–1100 nm was determined for each sample and samples with the same or similar percentage transmittances were used on solar cells for comparative experiments.

Sheet resistance measurements were performed on nanotube films using a four point probe attached to Keithlink software (Solon, OH, USA), three sets of five measurements were performed on each film at different orientations and the results were averaged. The resistance was measured at each stage of the solar cell treatment process. However, as HF etches glass, a 2% HCl treatment was performed for 10 s as per the HF treatment. Scanning electron microscopy (SEM) was performed using an FEI Inspect F50 (Hillsboro, OR, USA) on nanotube films mounted on silicon wafers.

Raman spectra of the various nanotube types were obtained using a Witec Alpha R confocal Raman microscope (Ulm, Germany). The laser used was a Nd:YAG 532 nm (2.33 eV) laser and the power used at the sample was less than 10 mW. A 40× magnification objective with a Numerical Aperture (NA) of 0.6 was used. The data collected were single spectra with 10 spectra collected per sample at 5 different locations on each sample. The integration time was between 5 and 10 s with 3 accumulations per spectrum.

## 5. Conclusions

Suspensions of each nanotube type were produced under the exact same solvent conditions and procedures. This allowed an unambiguous comparison to be made between different nanotube types in a silicon/CNT heterojunction solar cell in which the suspension and film preparation were identical.

It was observed that although one SWCNT sample produced a higher efficiency than the other samples, both single-walled samples and the double-walled sample produced similar power conversion efficiencies as each other, with a difference of less than 0.5% between the three samples. Single-walled films produced better short circuit current densities, whereas double-walled and multi-walled carbon nanotube films displayed higher open circuit voltages. Fill factors were found to be similar across the board, with SWCNT films producing better *R*_Shunt_ to *R*_Series_ ratios. It was discovered that cells made with MWCNT films improve to a lesser extent with doping treatment. Overall, the results of this study indicate that when variables other than nanotube type are controlled, large diameter single-walled carbon nanotube films provide the best performance in silicon/CNT solar cells. This conclusion differs from some previous work in which other variables were not well controlled, and will inform future research and development in this field.

## Figures and Tables

**Table 1:** D band to G band ratios for each nanotube type (two single-walled carbon nanotube samples (SWCNT-1 and SWCNT-2), one double-walled carbon nanotube sample (DWCNT) and one multi-walled carbon nanotube sample (MWCNT)).

Nanotube Sample	SWCNT-1	SWCNT-2	DWCNT	MWCNT
D/G Ratio	0.049	0.171	0.078	1.55

**Table 2:** Calculation of nanotube diameter from radial breathing mode (RBM) Raman shift.

Nanotube Sample	RBM Raman Shift (cm^−1^)	Calculated Diameter (nm)	Supplied Diameter (nm)
SWCNT-1	177	1.40	1.4–1.5
SWCNT-2	191, 239, 276	1.29, 1.02, 0.88	0.8–1.2
DWCNT Small Peaks	115, 125	2.03, 1.87	2–4
DWCNT Large Peaks	154, 186	1.52, 1.25	2–4

**Table 3:** Average sheet resistance for each carbon nanotube (CNT) sample with treatment (Ω·sq^−1^).

Film Type with Transmittance Percentage	As Prepared (Ω·sq^−1^)	HCl Treatment 1 (Ω·sq^−1^)	Thionyl Chloride Treatment (Ω·sq^−1^)	HCl Treatment 2 (Ω·sq^−1^)
SWCNT-1 60%	1440 ± 8.2%	951 ± 1.9%	693 ± 42%	543 ± 2.9%
SWCNT-2 65%	4070 ± 7.0%	3650 ± 2.6%	1880 ± 4.0%	2410 ± 12%
DWCNT 58%	138,000 ± 84%	4190 ± 39%	19,600 ± 138%	2550 ± 38%
MWCNT 60%	3340 ± 6.0%	3520 ± 6.0%	3020 ± 23%	2890 ± 27%

**Table 4:** Solar cell properties for best performing cells for each CNT sample in bold text, average properties and error values for sets of three (two for the Carbon Allotropes DWCNT) cells in regular text. *J*_SC_: Short circuit current density. *V*_OC_: Open circuit voltage. PCE: Power conversion efficiency. FF: fill factor.

	SWCNT-1	SWCNT-2	DWCNT	MWCNT
*J*_SC_ (mA cm^−2^)	**24.7;** 24 ± 4.3%	**22.5;** 22.2 ± 1.5%	**19.5;** 18.9 ± 4.5%	**18.4;** 18.1 ± 2.5%
*V*_OC_ (V)	**0.468;** 0.427 ± 9.17%	**0.483;** 0.449 ± 7.8%	**0.58;** 0.553 ± 1.3%	**0.533;** 0.465 ± 17.6%
FF	**0.41;** 0.41 ± 8.64%	**0.41;** 0.39 ± 5.13%	**0.40;** 0.40 ± 0.0%	**0.38;** 0.35 ± 8.7%
PCE%	**4.79;** 4.21 ± 18.74%	**4.45;** 3.90 ± 13.8%	**4.31;** 4.14 ± 6.0%	**3.78;** 3.03 ± 28.2%
*R*_shunt_ (Ohm)	**6.42 × 10^3^;** 2.81 × 10^3^ ± 112%	**1.06 × 10^3^;** 7.91 × 10^2^ ± 30.2%	**8.42 × 10^2^;** 9.61 × 10^2^ ± 17.5%	**8.07 × 10^2^;** 6.65 × 10^2^ ± 24.8%
*R*_series_ (Ohm)	**1.21 × 10^2^;** 1.08 × 10^2^ ± 16.3%	**1.11 × 10^2^;** 1.12 × 10^2^ ± 9.4%	**1.44 × 10^2^;** 1.57 × 10^2^ ± 11.3%	**1.64 × 10^2^;** 1.68 × 10^2^ ± 2.1%
Diode Ideality	**2.37;** 3.27 ± 23.96%	**1.98;** 1.95 ± 16.4%	**1.67;** 1.93 ± 19.3%	**1.94;** 2.75 ± 33.2%
*J*_SAT_ (mA cm^−2^)	**2.20 × 10^−3^;** 2.45 × 10^−1^ ± 106%	**4.64 × 10^−4^;** 1.81 × 10^−3^ ± 145%	**1.35 × 10^−5^;** 1.26 × 10^−4^ ± 126%	**1.31 × 10^−4^;** 1.39 × 10^−1^ ± 171%
Film Transmittance (%)	60	65	58	60
Final Sheet Resistance of Film (Ω·sq^−1^)	543 ± 2.9%	2410 ± 12%	2550 ± 38%	2890 ± 27%

**Table 5:** Types of nanotube samples used.

Type	Company	Diameter (nm)	Length (nm)	Purity (%)
SWCNT-1	Carbon Solutions (Riverside, CA, USA)	1.4–1.5	500–1500	90
SWCNT-2	NanoIntegris (Boisbriand, QC, Canada)	0.8–1.2	100–1000	95
DWCNT-1	Carbon Allotropes ((Kensington, NSW, Australia)	2–4	<1500	>>60
DWCNT-2	Sigma-Aldrich (St Louis, MI, USA)	3.5	3000	90
MWCNT	Sigma-Aldrich	9.5	1500	95

## Supplementary Materials

The following are available online at http://www.mdpi.com/2079-4991/6/3/52/s1, Figure S1: Dark J/V curves for cells for each type of sample after the second HF etch: (**a**) SWCNT-1; (**b**) SWCNT-2; (**c**) DWCNT; and (**d**) Sigma Aldrich MWCNT. Figure S2: A plot of the relation between *J*_SAT_ and *V*_OC_ for the best performing cells for each sample. Table S1: Sheet resistance as a function of thickness. The values marked with an asterisk were the volumes used to produce films for solar cells in this study.

## Abbreviations

The following abbreviations are used in this manuscript:

SWCNT: Single-walled Carbon Nanotube
DWCNT: Double-walled Carbon Nanotube
MWCNT: Multi-walled Carbon Nanotube
CNT: Carbon Nanotube
PCE: power conversion efficiency
CVD: chemical vapor deposition
PV: photovoltaics
RBM: radial breathing mode
