# Supplementary material for: Investigating the Effect of Carbon Nanotube Diameter and Wall Number in Carbon Nanotube/Silicon Heterojunction Solar Cells
Source: Nanomaterials (Basel). 2016 Mar 22;6(3):52. doi: 10.3390/nano6030052 (PMC5302527; doi:10.3390/nano6030052)
Supplement: Supplementary file 1 [file nanomaterials-06-00052-s001.pdf]

Supplementary Materials: Investigations into the Effect of Numbers of Carbon Nanotube Walls in Carbon Nanotube/Silicon Heterojunction Solar Cells

Tom Grace [1], LePing Yu [1], Christopher Gibson [1], Daniel Tune [1,2], Huda Alturaif [3], Zeid Al Othman [3] and Joseph Shapter [1,*]

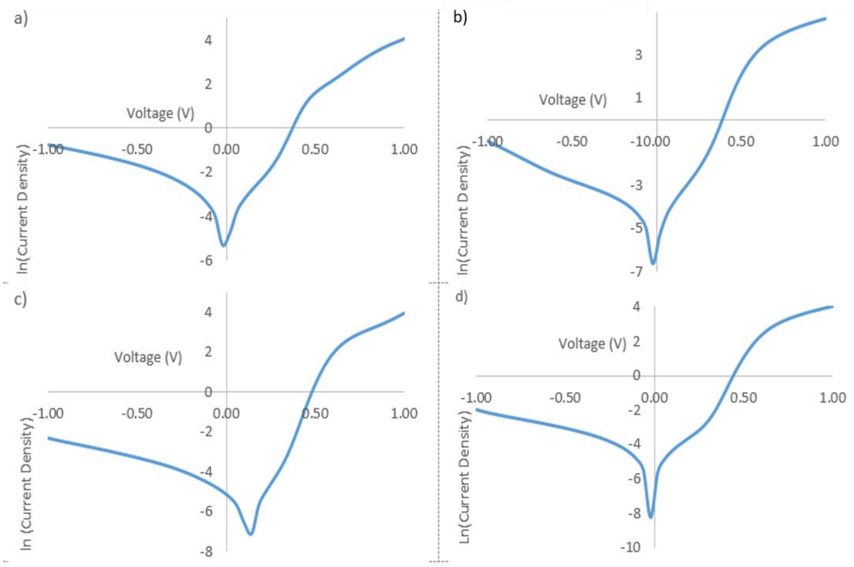

Figure S1. Current density *versus* voltage curves without illumination (Dark *J*/*V* curves) for cells for each type of sample after the second hydrofluoric acid (HF) etch: (**a**) single-walled carbon nanotube sample 1 (SWCNT–1); (**b**) single-walled carbon nanotube sample 2 (SWCNT–2); (**c**) double-walled carbon nanotube sample 1 (DWCNT); and (**d**) Sigma Aldrich (St Louis, MI, USA) multi-walled carbon nanotube (MWCNT).

Table S1. Sheet resistance and direct current (DC) electrical to optical conductivity, (σ_{DC}/σ_{OP}) as a function of thickness. The values marked with an asterisk were the volumes used to produce films for solar cells in this study. Samples measured were single-walled carbon nanotube sample 1 (SWCNT–1); single-walled carbon nanotube sample 2 (SWCNT–2); double-walled carbon nanotube sample 1 (DWCNT); and Sigma Aldrich multi-walled carbon nanotube (MWCNT).

Nanotube Type	Volume of Suspension (mL)	Transmittance (%)	Sheet Resistance (Ω sq^{-1})	DC/OP Conductivity
SWCNT–1	0.5	82	4060	0.445
	1.0	74	1200	0.967
	1.5	88	690	4.139
	2.0 *	56	530	1.058
	2.5	59	390	1.601
SWCNT–2	0.5	57	2,380,000	0.000
	1.0	85	4280	0.520
	1.5	64	4320	0.175
	2.0	65	2920	0.269
	2.5 *	57	2410	0.241
DWCNT–1	5.0	56	414,900	0.001
	7.5 *	55	2550	0.212
	10.0	56	4260	0.132
	12.5	41	17,200	0.020

MWCNT	1.0	62	4510	0.155
	1.5	72	4620	0.229
	2.0	65	4000	0.196
	2.5 *	52	2890	0.169
	3.0	52	3210	0.152

The ratio of the direct current (DC) electrical to optical conductivity, (σ_{DC}/σ_{OP}) was calculated as per Hecht *et al*. [1].

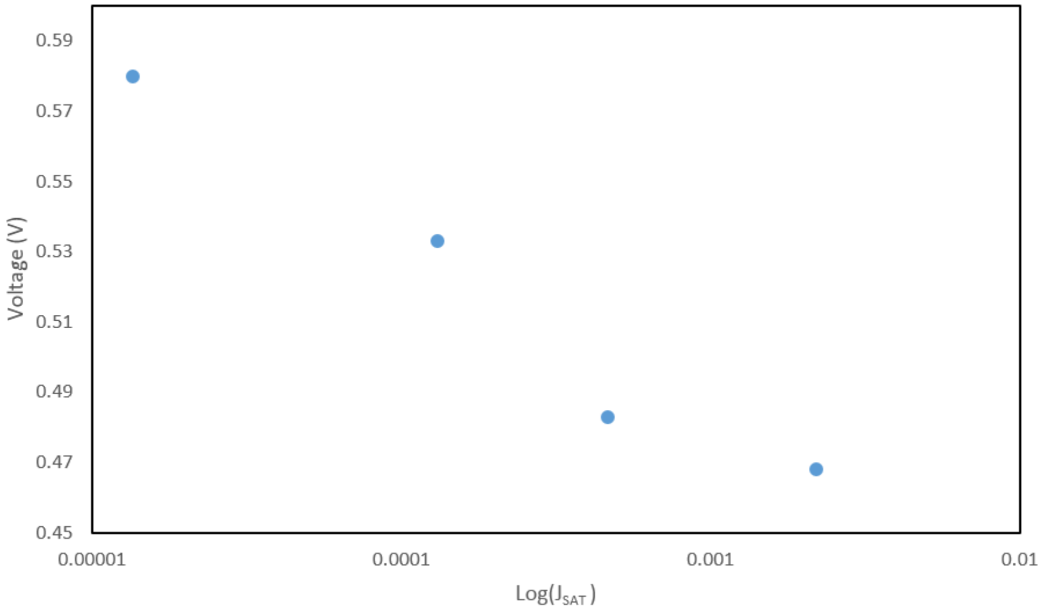

Figure S2. A plot of the relation between saturation current (J_{SAT}) and open circuit voltage (V_{OC}) for the best performing cells for each sample.

References

1. Hecht, D.S.; Heintz, A.M.; Lee, R.; Hu, L.; Moore, B.; Cucksey, C.; Risser, S. High conductivity transparent carbon nanotube films deposited from superacid. *Nanotechnology* **2011**, *22*, doi:10.1088/0957-4484/22/7/075201.

© 2016 by the authors; licensee MDPI, Basel, Switzerland. This article is an open access article distributed under the terms and conditions of the Creative Commons by Attribution (CC-BY) license (http://creativecommons.org/licenses/by/4.0/).
